# The Fungus *Aspergillus aculeatus* Enhances Salt-Stress Tolerance, Metabolite Accumulation, and Improves Forage Quality in Perennial Ryegrass

**DOI:** 10.3389/fmicb.2017.01664

**Published:** 2017-09-04

**Authors:** Xiaoning Li, Shijuan Han, Guangyang Wang, Xiaoying Liu, Erick Amombo, Yan Xie, Jinmin Fu

**Affiliations:** ^1^Key Laboratory of Plant Germplasm Enhancement and Specialty Agriculture, Wuhan Botanical Garden, Chinese Academy of Sciences Wuhan City, China; ^2^University of Chinese Academy of Sciences Beijing, China; ^3^School of Resources and Environmental Engineering, Ludong University Yantai, China

**Keywords:** perennial ryegrass, salt stress, *Aspergillus aculeatus*, physiological markers, forage quality, metabolites

## Abstract

Perennial ryegrass (*Lolium perenne*) is an important forage grass with high yield and superior quality in temperate regions which is widely used in parks, sport field, and other places. However, perennial ryegrass is moderately tolerant to salinity stress compared to other commercial cultivars and salt stress reduces their growth and productivity. *Aspergillus aculeatus* has been documented to participate in alleviating damage induced by salinity. Therefore, the objective of this study was to investigate the mechanisms underlying *A. aculeatus*-mediated salt tolerance, and forage quality of perennial ryegrass exposed to 0, 200, and 400 mM NaCl concentrations. Physiological markers and forage quality of perennial ryegrass to salt stress were evaluated based on the growth rate, photosynthesis, antioxidant enzymes activity, lipid peroxidation, ionic homeostasis, the nutritional value of forage, and metabolites. Plants inoculated with *A. aculeatus* exhibited higher relative growth rate (RGR), turf and forage quality under salt stress than un-inoculated plants. Moreover, in inoculated plants, the fungus remarkably improved plant photosynthetic efficiency, reduced the antioxidant enzymes activity (POD and CAT), and attenuated lipid peroxidation (decreased H_2_O_2_ and MDA accumulation) induced by salinity, compared to un-inoculated plants. Furthermore, the fungus also acts as an important role in maintaining the lower Na/K ratio and metabolites and lower the amino acids (Alanine, Proline, GABA, and Asparagine), and soluble sugars (Glucose and Fructose) for inoculated plants than un-inoculated ones. Our results suggest that *A. aculeatus* may be involved in modulating perennial ryegrass tolerance to salinity in various ways.

## Introduction

Salinity, one of the abiotic stresses, is a common and vital environmental factor that limits crop germination, growth, and productivity (Sairam et al., 2002). Salt stress can inhibit shoots and roots growth and perturb plant metabolism affecting their physiological status (Zhu, 2001). In addition, previous studies have shown that ~6% of land area around the world and 50% irrigated lands are acutely affected by salt stress (Rhoades and Loveday, 1990; Munns, 2005).

Salt can trigger osmotic stress through limiting water absorption from soil, and cause ionic stress as a result of excessive cellular accumulation of potentially toxic Na^+^ and Cl^−^ (Shannon, 1997; Kohler et al., 2009). Consistent to this finding, it has been emphasized that elevated salinity level affected forage quality parameters, such as crude protein, organic matter, and neutral detergent fiber (Robinson et al., 2004). Furthermore, increasing evidences have indicated that salinity was detrimental on photosynthesis as evinced by lower photosynthesis rate on salt-stressed plants (Allakhverdiev and Murata, 2008; Kalaji et al., 2011). In separate studies, it was reported that salt stress can also be associated with an oxidative stress as a result of the production of reactive oxygen species (ROS), thereby leading to negative impact on plant growth (Kohler et al., 2009). Fortunately, plants have evolved a series of antioxidant enzymes that can scavenge the ROS for self-protection in response to oxidative stress, such as peroxidase (POD), superoxide dismutase (SOD), and catalase (CAT) (Apel and Hirt, 2004).

Plants that are exposed to salt stress would elicit a series of metabolic responses (Widodo et al., 2009), and alteration of a wide range of metabolites were observed such as sugars, amino acids, and organic acids (Hu et al., 2014). In previous report, it was been demonstrated that soluble sugars (such as glucose, sucrose, and fructose) were sensitive to environmental stresses, and were highly accumulated in perennial ryegrass after salt treatment (Hu et al., 2013). Recently, it has been found that one of the primary strategies of plants response to salt stress is the accumulation of compatible solutes, such as free amino acids, and sugars (Hu et al., 2014; Araújo et al., 2015).

To better withstand the detrimental effect of salt stress, several remediation strategies for saline soil are feasible, such as growing salt-tolerant plants in saline soil, desalinating soil by leaching excessive salinity, and exploiting the symbiotic relationship between plants and microbes (Alkaraki et al., 2001; Feng et al., 2002; Bandou et al., 2006). Among those remediation methods, the desalination of saline soil is not economically viable for sustainable agriculture due to their adverse environmental effects (Einav et al., 2003; Bandou et al., 2006). Some investigations have demonstrated that abiotic-stress tolerance can be enhanced by microbes forming mutualistic interactions with the plants (Waller et al., 2005; Baltruschat et al., 2008). In particular, root endophyte *Piriformospora indica* were demonstrated to protect plants against salt stress, and conferred salt tolerance to *P. indica*-colonized plant (Waller et al., 2005; Baltruschat et al., 2008). Furthermore, microbes-mediated plant stress mitigation has occurred as a significant component in the process of plant managing salt stress, and their role in enhancing growth and productivity of plant has been well-established (Yang et al., 2009; Ruiz-Lozano et al., 2012). Several studies have evidenced that arbuscular mycorrhizal fungi is beneficial for plant growth and absorption of nutrients under adverse salinity conditions (Alkaraki et al., 2001; Bandou et al., 2006). In addition, enhancing the partnership between plants, and beneficial rhizosphere fungi could promote plant growth and improve plant biomass production (Xie et al., 2014b). Therefore, microbes have been considered as a kind of bio-ameliorator in saline soils.

Perennial ryegrass (*Lolium perenne* L.) is one of the most crucial forage grass species due to its high yield, good quality and high nutritive values in temperate regions (Wilkins, 1991). In addition, it is extensively used as a cold-season turf grass as a consequence of its dense root system, superior tillering, and regeneration ability (Hannaway et al., 1999). However, it has been demonstrated that the salinity tolerance of perennial ryegrass is ranked as moderate for commercial cultivars (Ali Harivandi et al., 1992). Therefore, enhancing the perennial ryegrass to better counter salt stress is very essential for improving its growth and production.

*Aspergillus aculeatus* as a Cd-resistant fungus that was isolated from the rhizosphere of bermudagrass. It has been evidenced that the *A. aculeatus* can colonize plant roots, improve turf quality, chlorophyll content and facilitate plant growth, and photosynthesis thereby alleviating toxic effects of cadmium on bermudagrass (Xie et al., 2014b). In addition, *A. aculeatus* can be easily cultured in axenic cultures, which enables easy propagation due to it has no host specificity. In our previous research, we have found that *A. aculeatus* has an important effect on attenuating salt stress by producing indole-3-acetic acid, and siderophores; thereby conferring stress tolerance to plants (Unpublished data). Furthermore, the previous study indicated that *A. aculeatus* could solubilize natural forms of phosphorus, and accelerate plant uptake and utilization of phosphorus (Narsian and Patel, 2000). However, little is known about the role of *A. aculeatus* in inducing perennial ryegrass resistance to salt stress. Therefore, the *A. aculeatus* might provide a high application value in perennial ryegrass adapting saline stress environments, and remediating salinity soil.

Based on the above study reports, here we investigated the effects of *A. aculeatus* on perennial ryegrass defense against salt stress. To expound on *A. aculeatus*-mediated protective mechanism of ryegrass against salt stress, we measured important indicators of salt stress, such as growth rate, nutritive values of perennial ryegrass; furthermore, we analyzed antioxidant activities activity, lipid peroxidation, photosynthetic performance, ionic homeostasis, and metabolic homeostasis.

## Materials and methods

### Activation and propagation of the fungi

The fungi for this experiment were screened and identified as *A. aculeatus* by Xie et al. (2014b) and were maintained in the refrigerator at −80°C. Prior to the experiment, the fungi were activated three times, and every activation process lasted for 48 h with Martin agar medium in a growth chamber at 30°C. The Martin agar medium: 0.5 g MgSO_4_·7H_2_O, 1 g KH_2_PO_4_, 5 g peptone, 10 g glucose, 18 g agar, diluted with distilled water to 1 L, adding 1% Rose Bengal sodium salt 3.3 mL (Xie et al., 2014b).

After fungal activation, for further propagation, the fungi were inoculated in the Martin liquid medium (0.5 g MgSO_4_·7H_2_O, 1 g KH_2_PO_4_, 5 g peptone, 10 g glucose, with the 1 L distilled water). Before mixing with the fungi, the liquid Martin medium was transferred to the 250 mL-Erlenmeyer flasks filled with 150 mL; and then all flasks were sterilized in an autoclave at 121°C for 20 min. After sterilization, sterilized streptomycin (1%) was added to the flasks (0.3/100 mL). Finally, all flasks were placed on a shaker at 200 × g and 30°C for 48 h.

### Preparation of the growth substances

The growth substances (1 sawdust: 3 sand, v/v, pH = 6.5) were divided into halves, where one was inoculated with fungi and the other was left un-inoculated. All growth substances were weighed and sterilized at 127°C in autoclave for 1 h; and then transferred into the plastic cups (15 cm in diameter and 20 cm deep). All the pots were pitted at the bottom to allow soil aeration and to drain excess water; and then were sterilized using UV for 1 h. To inoculate the fungi into the partial growth substances, inoculated fungi was filtered using gauze, and was washed three times using sterile water to rinse out all liquid Martin medium on the surface of fungi; and then transferred into the growth matrix and mixed thoroughly for cultivating 48 h in a growth chamber at 30°C with chamber at 30°C.

### Plant materials and growth conditions

The seeds of perennial ryegrass “lark” were used as plant material in this research. The seeds were surface-sterilized by 70% ethanol for 5 min, and then with 0.1% HgCl_2_ for 5 min and were washed five times using sterilized distilled water. Subsequently, the seeds were sowed evenly in plastic cups that were filled with pre-prepared growth substances (as described in Section Preparation of the Growth Substances), and covered by a layer of sand. After germination, the cups were watered daily, and fertilized twice weekly with half-strength Hoagland nutrient solution (Hoagland and Arnon, 1950). Thereafter, the materials were clipped at 8 cm above the growth substances surface. All cups were placed in a greenhouse with daily temperature of 21 ± 3/18 ± 3°C (day/night), a 14 h photoperiod. To establish the leaves and roots of plants, the seedlings were allowed to grow in the above-mentioned conditions for 6 weeks before the NaCl treatments. After establishment, all the pots were placed in the artificial intelligence incubator at a constant temperature of 14/10 h for light/dark, 24/22°C for day/night, 400 μmol photons m^−2^ s^−1^ of light intensity, 70 ± 10% relative humidity used here 2 weeks for adaptation before the salt treatment.

### Experimental design and treatment

After 2 weeks of adaptation, to determine the transpiration rate before the salt treatment, the plant-cup system was weighed at 24-h intervals through the water balance method described by previous report. Seedlings with similar transpiration rate were selected for each replicate of the salt treatments. The experimental materials, perennial ryegrass “lark,” were subjected to two treatments: NaCl and NaCl + *A. aculeatus* and three salinity concentration: 0 mM (control), 200, and 400 mM NaCl was dissolved in Hoagland nutrient solution. Salinity concentration was gradually stepped up as 50 mM increments at 24-h intervals until a final salinity concentration of 200 and 400 mM was achieved and the final concentration was maintained for 2 weeks. After the final salinity, the shoot was clipped to uniform height. During the period of salt treatment, the plants were supplied with sufficient water and nutrition. At the end of the experiment, the shoots, and roots lengths were measured to assess the RGR, and subsequently the leaves samples were harvested for measuring other parameters. The salinity treatment was arranged in a randomized and complete block design with five replicates.

### Measurements

#### Growth rate

In order to estimate the average relative growth rate (RGR) of the shoot, the difference in average turf canopy height before and after treatment was measured according to the method described by Hu et al. (2013). For the RGR of the root, the roots elongating before treatment and after treatment was measured. The RGR was calculated according to the following formula (Equation 1), Where H_*t*_ and H_0_ represent the height of final and initial measurement, respectively, and Δ*t* is the duration of the experiment (14 d) (Hu et al., 2013).

(1) $$\text{RGR}=(\text{In}H_{t}-\text{In}H_{0})/\Delta t$$

#### Electrolyte leakage

In order to determine cell membrane stability, the electrolyte leakage (EL) was measured according to the following method: 0.1 g the fully expanded leaves were collected and washed three times using deionized water; and then were immediately cut into uniform long segments (ca. 0.5 cm). Subsequently, the leaf pieces were submerged in 15 mL deionized water in a 50-mL centrifuge tube and shaken at 25°C for 24 h using a rotary shaker. Then, the initial electrical conductivity (Ci) was measured with a conductivity meter (JENCO-3173, Jenco Instruments, Inc., San Diego, CA, USA). Then, to completely release all the tissues electrolytes, the leaf tissues were killed by autoclaving for 30 min at 121°C. After the solution with killed tissues cooling to room temperature, the electrical conductivity (Cmax) was measured. EL was calculated according to the following formula:

(2) EL (%)=(Ci/Cmax)×100

#### Enzymes activity and lipid peroxidation

To determine the activity of antioxidant enzymes POD, CAT, and the content of the MDA, 0.3 g of fully expanded leaf samples were harvested, and immediately grounded into powder using pre-chilled mortar and pestle with liquid nitrogen. After that, the powder was homogenized in 4 mL ice-cold phosphate buffer (50 mM, pH 7.8) including 0.7% NaH_2_PO_4_·2H_2_O and 1.64% Na_2_HPO_4_·12 H_2_O. Then, the homogenates were transferred into 10 mL-centrifuge tubes and centrifuged at 12,000 rpm 20 min at 4°C. The supernatant was collected and stored for the determination of POD and CAT activity, MDA and H_2_O_2_ content.

The content of malondialdehyde (MDA), POD (EC 1.11.1.7), and CAT (EC 1.11.1.6) activity was assayed based on the method as described by Hu et al. (2011). The content of H_2_O_2_ was determined based on the method described by manufacturer protocols of hydrogen peroxide assay kit (Beyotime, S0038). The absorbance was immediately recorded immediately at 405 nm and was calculated according to the standard curve generated with known concentrations of H_2_O_2_.

#### Chlorophyll (Chl) a fluorescence transient and total organic carbon (TOC)

Chlorophyll a fluorescence transient (OJIP curve) was measured according to the method as described by Chen et al. (2013) with a pulse-amplitude modulation (PAM) fluorometer (PAM 2500, Heinz Walz GmbH). At the end of treatment, the fourth fully expanded leaves were collected, and pre-adapted in the dark for 30 min, which ensured that all reaction centers of PSII were closed thereby acquiring the maximal fluorescence intensity (F_M_). After adaption, the OJIP transients were monitored with a red light of 3,000 μmol photons m^−2^ s^−1^. The Chlorophyll a fluorescence emission triggered by strong light pulses was determined and digitized between 10 μs and 320 ms. In order to better analyzed the OJIP curve, the JIP-test was applied as Table 1.

**Table 1:** Photosynthetic parameters deduced by the JIP-test analysis of fluorescence transients in Figure 4.

	**CK**	**F**	**200 mM**	**200 mM + F**	**400 mM**	**400 mM + F**	**Definitions**
**DATA EXTRACTED FROM THE RECORDED OJIP FLUORESCENCE TRANSIENT CURVES**							
F_0_ = 20 μs	0.38a	0.38a	0.34a	0.36a	0.36a	0.32a	Fluorescence at time t after onset of actinic illumination
F_K_	0.90a	0.90a	0.80a	0.82ab	0.79a	0.76b	Fluorescence value at 300 μs
F_J_	1.04a	1.06a	0.97a	0.98b	0.90a	0.93b	Fluorescence value at the J-step of OJIP
F_I_	1.41a	1.41a	1.31a	1.34ab^*^	1.26a	1.31b	Fluorescence value at the I-step of OJIP
F_P_ = F_M_	1.52a	1.58a	1.43ab	1.47b^*^	1.38b	1.43b^*^	Fluorescence value at the peak of OJIP test
V_J_	0.58a	0.57a	0.58a	0.56a	0.55a	0.55a	Relative variable fluorescence at the J-step
V_I_	0.90a	0.86b	0.88a	0.88a	0.92a	0.90a^*^	Relative variable fluorescence at the I-step
Mo	1.80a	1.75a	1.67a	1.66a	1.75a	1.59a	Approximate value of the initial slope of fluorescence transient curves
**QUANTUM YIELDS AND EFFICIENCIES/PROBABILITIES**							
φPo	0.75a	0.76a	0.76a	0.76a	0.73a	0.77a^*^	Maximum quantum yield for primary photochemistry, namely F_V_ /F_M_
φEo	0.32a	0.33a	0.32a	0.34a	0.33a	0.34a^*^	Quantum yield of the electron transport flux from Q_A_ to Q_B_
φRo	0.07a	0.11a	0.09a	0.09b	0.06a	0.08b^*^	Quantum yield for reduction of end electron acceptors at the PSI acceptor side
ΨEo	0.42a	0.43a	0.42a	0.44a	0.45a	0.45a	Efficiency/probability with which a PSII trapped electron is transferred from Q_A_ to Q_B_
δRo	0.22a	0.33a	0.27a	0.26b	0.18a	0.23c^*^	Efficiency/probability with which an electron from Q_B_ is transferred until PSI acceptors
**SPECIFIC ENERGY FLUXES (PER ACTIVE PSII REACTION CENTER)**							
ABS/RC	0.78a	0.76a	0.73bc	0.70b	0.71c	0.68b	Absorbed photon flux per RC
γRC2	0.20a	0.20a	0.21a	0.20a	0.19a	0.21a^*^	Probability that a PSII Chl molecule functions as RC
RC/ABS	1.31a	1.32b	1.37a	1.43ab	1.42a	1.47a	Number of Q_A_ reducing RCs per PSII antenna Chl
TP_0_/RC	3.09a	3.07a	2.90a	2.98a	3.18a	2.86a	Trapped excitation flux (leading to Q_A_ reduction) per RC
ET_0_/RC	1.34b	1.35a	1.23b	1.31a	1.43a	1.27a^*^	Electron transport flux (further than ${\text{Q}}_{\text{A}}^{-}$ per RC
RE_0_/RC	0.29a	0.43a	0.34a	0.35b	0.25a	0.30c^*^	Electron flux reducing end electron acceptors at the PSI acceptor side, per RC
**PERFORMANCE INDEXES (PI**, **COMBINATION OF PARAMETERS)**							
PI_ABS_	0.57a	0.60a	0.62a	0.63a	0.53a	0.75a^*^	PI (potential) for energy conservation from exciton to the reduction of intersystem electron
PI_total_	0.18a	0.30a	0.23a	0.23a	0.12a	0.23a^*^	PI(potential) for energy conservation from exciton to the reduction of PSI end acceptors

*F represents A. aculeatus. The NaCl concentration is 0, 200, and 400 mM and the numbers marked with the same small letter indicate insignificant differences under only salt treatment or NaCl + A. aculeatus treatment with the different salt concentrations (P < 0.05). The asterisk indicate significant differences under salt treatment and NaCl + A. aculeatus treatment with the same salt concentrations (P < 0.05)*.

The total organic C was measured using a Stable Isotope Mass Spectrometer (Delta V Advantage, Thermo Finnigan, Germany). The oven-dried leaves were grounded into powder and weighed (0.3–0.4 mg) and then transferred into tin capsules with carbamide regarded as reference.

#### Ion content and forage nutritive value

At the end of the experiment treatment, all plant leaves were excised and were washed carefully with deionized water. Subsequently, all samples were put into the oven for 30 min at 105°C to deactivate the enzymes and dried for 48 h at 80°C. Then, the dried samples were finely ground with multichannel tissue ball milling apparatus (Scientz-192, Scientz biotechnology, GmbH, Ning Bo, China) and the individual sample was weighed 0.1 g. Samples were digested in 5 mL 66% HNO_3_ and 1 mL 30% H_2_O_2_ using a Microwave Sample Preparation System (ETHOS ONE, Milestone) with digestion procedure: 130°C for 12 min, 160°C for 8 min, and finally 160°C for 30 min. The mineral extract was transferred to 50 mL-volumetric flask and then filtrated using 0.45 μm filter membrane. The ion concentration was defined with inductively coupled plasma optical emission spectroscopy (ICP-OES, OPTIMA 8000DV, Perkin Elmer, USA).

Crude protein (CP) was evaluated according to the Kjeldahl method (AOAC, 1999) and crude fat (CF) was measured by extracting sample in petroleum ether using the Soxherm apparatus based on the Soxhlet method (AOAC, 1990). Forage P content was determined according to the Mo-Sb colorimetric method (Cao et al., 2015). Neutral detergent fiber (NDF) and acid detergent fiber (ADF) were performed as described in the previous report (Palmonari et al., 2014).

#### Metabolites extraction and derivatization

For the metabolite assay, fully expanded perennial ryegrass leaves (about 0.3 g) was harvested after experimental treatment, and frozen immediately in liquid nitrogen then stored in the refrigerator at −80°C until further analysis. The metabolite extraction and sample derivatization were extracted according to the protocol as described previously (Xie et al., 2014a). The frozen plant samples were ground into a fine powder in liquid nitrogen with pre-chilled mortar and pestle, then transferred powder into a 2-mL centrifuge tube containing 4.2 mL of 80% (v/v) aqueous methanol. Subsequently, the tubes were shaken for 2 h at 200 × g at ambient temperature, and then 60 μL of ribitol (2 mg mL^−1^) was added into the solution as internal standard. After that, the solution was heated in a water bath at 70°C for 15 min and centrifuged for 15 min at 12,000 × g, and then the supernatant was transferred into a new 10-mL centrifuge tube containing 4.5 mL of deionized water and 2.25 mL of chloroform. The mixture solution was vortexed fully for 15 s and centrifuged at 10,000 g for 10 min. The supernatant (i.e., polar phase) 0.3 mL was transferred into 2 mL HPLC vials and then was dried via using a centrifugal concentrator at 900 × g for overnight (Labogene, Denmark). The dried polar phase was derivatizated with 80 mL of methoxyamine hydrochloride (20 mg ml^−1^) dissolved in pyridine at 30°C for 2 h and was trimethylsilylated with 50 μL N-Methyl-N-(trimethylsilyl) trifluoroacetamide (MSTFA) for 2 h at 30°C. The reagents used in our research were purchased from Sigma-Aldrich Co. Ltd. (Poole, UK).

#### Gas chromatography-mass spectrometry (GC-MS) analysis

The metabolites were measured using a GC-MS (gas chromatography-mass spectrometry, Agilent 7890A/5975C, Agilent Technologies, Palo Alto, CA, USA) analysis based on the protocol of Xie et al. (2014a). For GC-MS, the derivatizated samples (1 μL) was injected into a DB-5MS capillary (30 m × 0.25 mm × 0.25 μm, Agilent J&W GC column, USA). The procedure was performed as follows: the inlet temperature was set at 280°C and 5-min solvent delay. Subsequently, the initial GC oven temperature was set at 70°C; 1 min after injection, the temperature of GC oven was increased to 280°C with 5°C per min, and then kept at 280°C for 10 min. The injection temperature was set at 280°C, while the ion source temperature was adjusted to 230°C. Helium was utilized as the carrier gas with a constant flow rate set at 1 mL per min. The measurement was recorded at two scans s^−1^ with 70 eV of electron impact ionization in a full scan mode (m/z range: 30–650).

#### Metabolite data processing and analysis

The metabolites were identified according to the retention time using software (Agilent MSD Productivity ChemStation) and compared with the reference spectra in commercially available compound libraries (NIST 11) (Gaithersburg, MD, USA). After the metabolites identification, relative quantification of the metabolite was performed according to the pre-added ribitol as the internal standard in the process of extraction of metabolites. Using the MetaboAnalyst webpage (http://www.metaboanalyst.ca/MetaboAnalyst/), the hierarchical clustering analysis (HCA) and principal component analysis (PCA) was assessed. The log-transformed response ratios of individual identified metabolite were calculated before statistical assessment.

### Statistical analysis

All mean data were based on analysis of variance using SPSS 20.0. Significant differences between means were performed combining one-way ANOVAs and Student-Newman–Keuls test. Differences were significant at *P* < 0.05.

## Results

### Effects of *A. aculeatus* on growth and physiological properties of perennial ryegrass under salt stress

**Figure 1:**
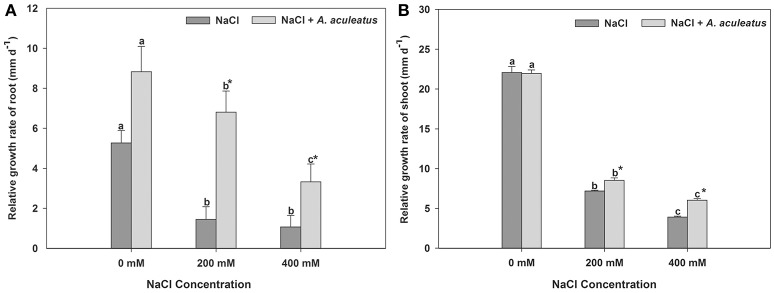
Influences of the *A. aculeatus* on relative growth rate of perennial ryegrass root **(A)** and shoot **(B)** under salt stress. Columns marked with same small letter indicate insignificant differences under only NaCl treatment or NaCl + *A. aculeatus* treatment with the different salt concentrations (*P* < 0.05). Columns marked with asterisk indicate significant differences under NaCl treatment and NaCl + *A. aculeatus* treatment with the same salt concentrations (*P* < 0.05).

The salt treatment dramatically reduced plant root and shoot RGR when compared with the control, regardless of *A. aculeatus* application (Figure 1). However, compared to un-inoculated plants, *A. aculeatus*-inoculated plants exhibited a significant increase in the RGR under salt stress (200 and 400 mM NaCl) (Figure 1). Interestingly, compared to control (0 mM NaCl), the RGR of the root was profoundly increased by *A. aculeatus* (Figure 1A).

**Figure 2:**
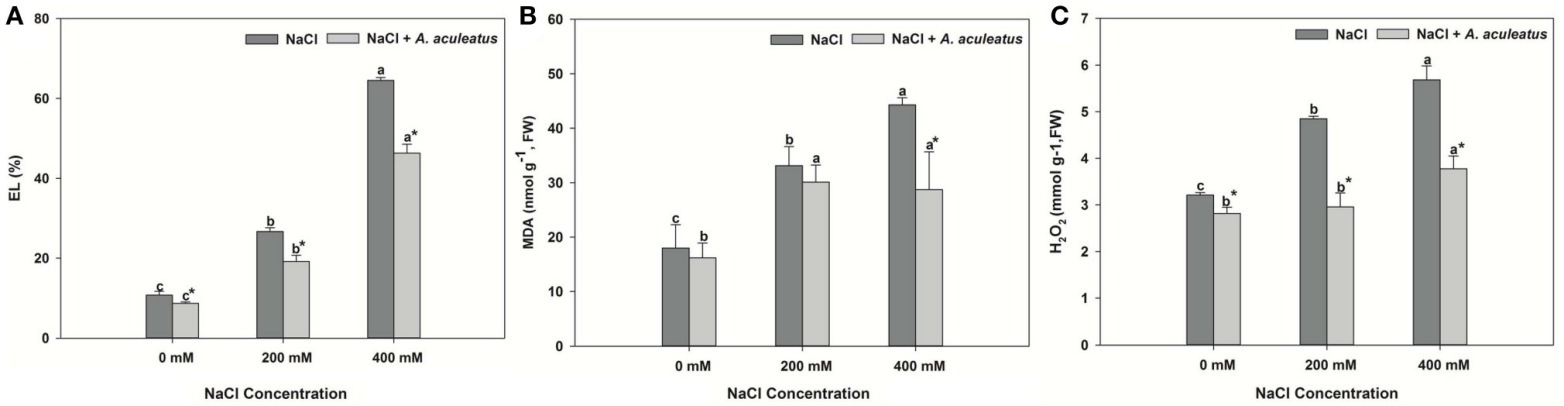
Electrolyte leakage (EL) **(A)**, malondialdehyde (MDA) **(B)**, and hydrogen peroxide (H_2_O_2_) **(C)** accumulation in leaves of perennial ryegrass under salt stress. Columns marked with same small letter indicate insignificant differences under only NaCl treatment or NaCl + *A. aculeatus* treatment with the different salt concentrations (*P* < 0.05). Columns marked with asterisk indicate significant differences under NaCl treatment and NaCl + *A. aculeatus* treatment with the same salt concentrations (*P* < 0.05).

The leaves of perennial ryegrass displayed higher salt-induced EL level, MDA content and H_2_O_2_ accumulation than those of control (Figures 2A–C). Obviously, inoculation of *A. aculeatus* markedly decreased the EL level and H_2_O_2_ production, compared to non-inoculated plants regardless of NaCl level, but the MDA content remarkably reduced by *A. aculeatu*s only under 400 mM NaCl, compared to un-inoculated plant.

**Figure 3:**
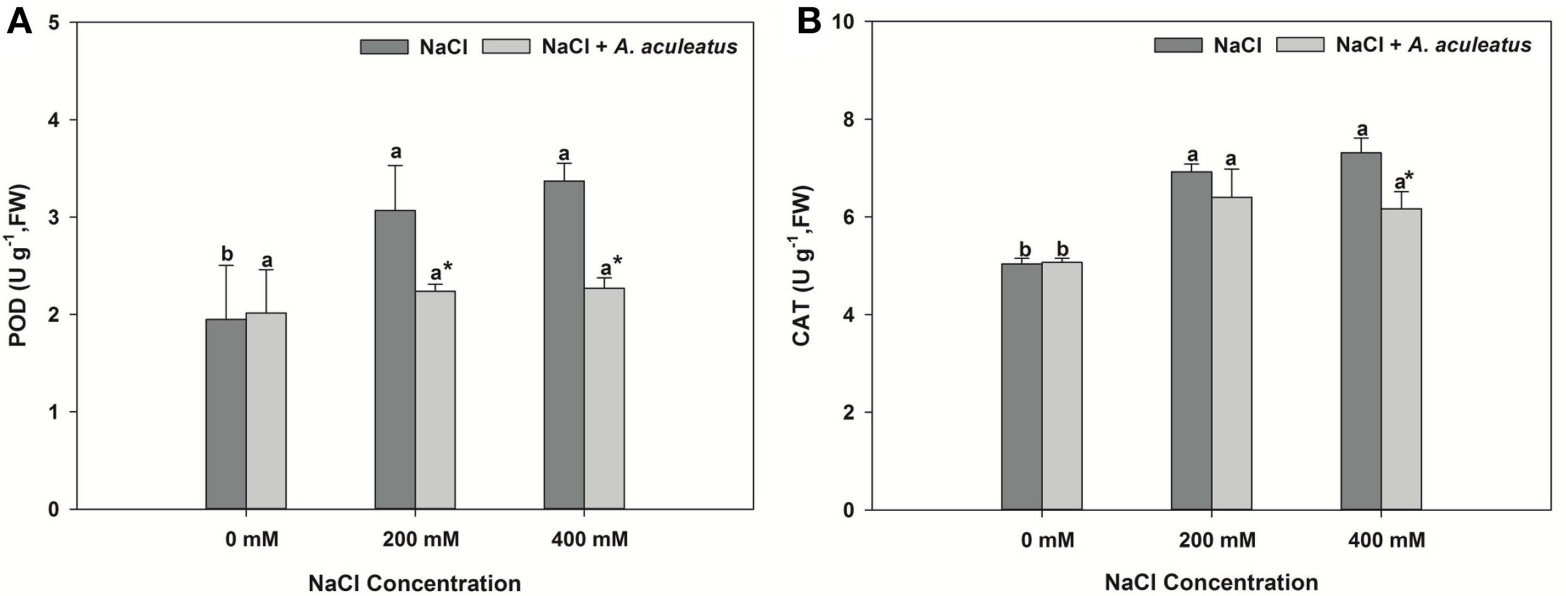
Peroxidase (POD) **(A)** and catalase (CAT) **(B)** content in leaves of perennial ryegrass exposed to salt stress. Columns marked with same small letter indicate insignificant differences under only NaCl treatment or NaCl + *A. aculeatus* treatment with the different salt concentrations (*P* < 0.05). Columns marked with asterisk indicate significant differences under NaCl treatment and NaCl + *A. aculeatus* treatment with the same salt concentrations (*P* < 0.05).

The salt treatment alone greatly enhanced leaf POD and CAT activity when compared to non-salinity conditions. Application of *A. aculeatus* markedly reduced the POD and CAT (only 400 mM salt concentration) activity compared to non-fungi treatment when plant subjected to salt stress (Figures 3A,B).

### Effects of *A. aculeatus* on photosynthetic efficiency of perennial ryegrass under salt stress

**Figure 4:**
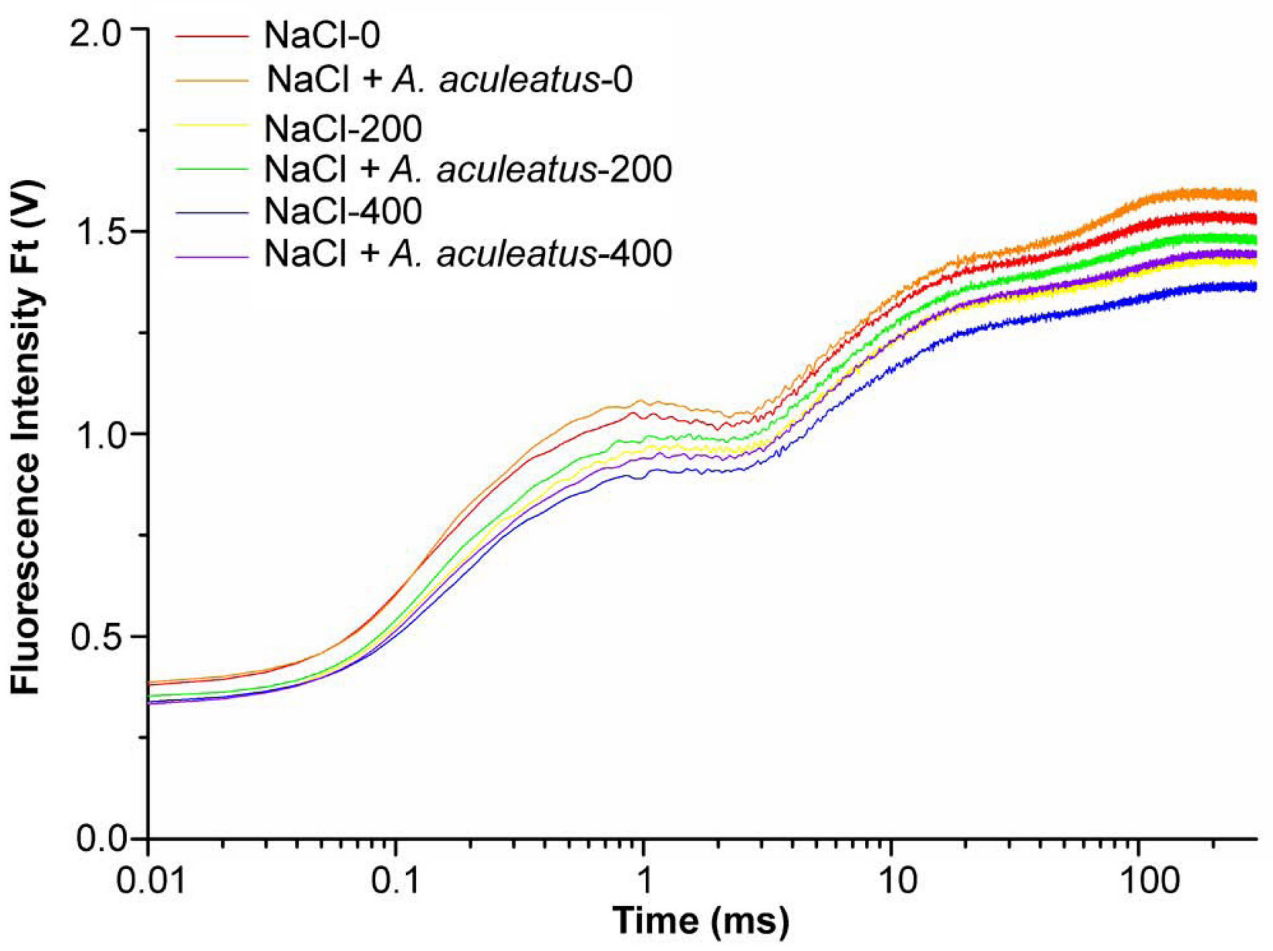
Alterations of chlorophyll fluorescence transients (OJIP curve) in leaves of perennial ryegrass grown with or without *A. aculeatus* under 0, 200, 400 mM NaCl stress.

Salt as well as a combination of *A. aculeatus* and salt treatment significantly affected the OJIP fluorescence transient curves of perennial ryegrass leaves (Figure 4), According to the results, the OJIP transient curve in plant leaves was higher under control than those under only salt stress, and the curve reduced with the increase of salt concentration. Furthermore, compared to non-inoculated, *A. aculeatus*-inoculated perennial ryegrass exhibited a higher OJIP fluorescence transient curve regardless of NaCl level (Figure 4).

To further investigate the effect of *A. aculeatus* on photosynthetic behavior in perennial ryegrass leaves exposed to salt stress, the JIP-test was used to explore the OJIP fluorescence transient curves. The value of basic fluorescence parameters was extracted from the recorded OJIP curves and multiple structural and functional parameters were calculated and analyzed. As shown in Table 1, basic parameters including F_0_, F_K_, F_J_, F_I_, F_M_, V_J_, and M_0_ displayed a down-regulated trend when plants were subjected to salt treatment, compared to control level. However, there was no significant difference between control and salt treatment expect for the F_M_ which remarkably declined under 400 mM NaCl treatment compared to control. Interestingly, compared to non-inoculated plants, *A. aculeatus*-inoculated regime had a higher value of F_I_ and F_M_, under salt stress.

The parameters of quantum yields and efficiencies, (such as φP_0_, φE_0_, φR_0_, δR_0_) were higher in *A. aculeatus*-inoculated perennial ryegrass leaves than non-inoculated ones subjected to 400 mM NaCl concentration. Moreover, the specific energy fluxes including values of ABS/RC, γRC2, RC/ABS, TP_0_/RC, ET_0_/RC, and RE_0_/RC exhibited differential changes against different treatments. The Table 1 showed that the salt stress decreased profoundly the value of ABS/RC and RE_0_/RC, however, other parameters had no obvious change when compared to control condition. Interestingly, the values of γRC2 and RE_0_/RC significantly increased, and ET_0_/RC dramatically declined in plants inoculated with *A. aculeatus* compared to non-inoculated ones exposed to 400 mM NaCl treatment. Additionally, PI_ABS_ and PI_total_ play a vital role in describing the overall activity of PSII. There was no remarkable difference between the control and salt stress. However, when plants subjected to 400 mM NaCl, while *A. aculeatus*-inoculated had higher values of PI_ABS_ and PI_total_ compared to non-inoculated plants (Table 1).

### Effects of *A. aculeatus* on ionic homeostasis and forage nutritive value of perennial ryegrass under salt stress

**Figure 5:**
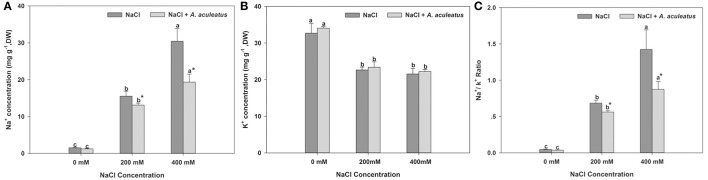
Influences of the *A. aculeatus* on Na^+^ concentration **(A)**, K^+^ concentration **(B)**, and Na^+^/ K^+^ ratio **(C)** under salt stress. Columns marked with same small letter indicate insignificant differences under only NaCl treatment or NaCl + *A. aculeatus* treatment with the different salt concentrations (*P* < 0.05). Columns marked with asterisk indicate significant differences under NaCl treatment and NaCl + *A. aculeatus* treatment with the same salt concentrations (*P* < 0.05).

Salt stress triggered a distinct accumulation of Na^+^ content in perennial ryegrass leaves compared to the non-salinity regime. However, in *A. aculeatus*-inoculated plant leaves, the application of fungi significantly decreased the Na^+^ accumulation compared to those non-inoculated plants under salt stress. Furthermore, the salt stress remarkably declined the K^+^ accumulation in leaves compared to control. However, in plants inoculated with fungi, the K^+^ content slightly increased but not significantly compared to non-inoculated ones against salt stress. Compared with control, salt regime dramatically increased Na^+^/K^+^ ratio in plants leaves. By contrast, the addition of *A. aculeatus* in fungi-inoculated plant leaves obviously decreased the Na^+^/K^+^ ratio, compared to non-fungi leaves when exposed to salt stress (Figure 5).

As shown in Table 2, high salt stress (400 mM NaCl concentration) significantly decreased the content of CF, P, NDF, and ADF, but slightly reduced in CP content, compared to non-salinity regime. However, inoculated plant had higher CP, CF, and P than un-inoculated plant, regardless of NaCl stress. At the same time, there were no significant differences in the NDF and ADF contents between un-inoculated and inoculated plant.

**Table 2:** Influence of the *Aspergillus aculeatus* on forage quality of perennial ryegrass exposed to 0, 200, and 400 mM NaCl concentration.

**Forage quality (%)**	**Treatment (0, 200, and 400 mM NaCl concentration)**					
	**CK**	**F**	**200 mM**	**200 mM + F**	**400 mM**	**400 mM+ F**
CP	18.64 ± 1.15a	21.96 ± 0.09a^*^	18.62 ± 0.23a	22.01 ± 0.49a^*^	18.31 ± 0.33a	19.96 ± 0.62b^*^
CF	9.83 ± 0.20a	11.95 ± 0.69a^*^	9.97 ± 0.53a	10.37 ± 0.85b	8.80 ± 0.57b	9.72 ± 0.28b
P	0.40 ± 0.01a	0.54 ± 0.02a^*^	0.35 ± 0.01b	0.47 ± 0.01b^*^	0.31 ± 0.01c	0.40 ± 0.01c^*^
NDF	50.79 ± 0.89a	51.02 ± 0.34a	45.72 ± 0.92b	42.98 ± 1.06b	43.88 ± 1.39b	41.88 ± 2.96b
ADF	26.89 ± 1.14a	25.57 ± 0.91a	23.32 ± 0.53b	21.98 ± 0.71b	23.04 ± 0.19b	21.34 ± 0.50b

*F represents A. aculeatus. NaCl concentration is 0, 200, and 400 mM and the results shown are the mean ± SD. The CP, CF, P, NDF, and ADF, respectively, represented the content of crude protein, crude fat, phosphorus, neutral detergent fiber, and acid detergent fiber. The numbers marked with the same small letter indicate no significant differences for comparison of treatments based on SNK test (P < 0.05). The asterisk indicate significant differences under salt treatment and NaCl + A. aculeatus treatment with the same salt concentrations (P < 0.05)*.

### Effects of *A. aculeatus* on metabolic homeostasis of perennial ryegrass under salt stress

To explore the metabolic homeostasis induced by exogenous fungi, *A. aculeatus*, under salt stress (400 mM NaCl concentration), GC-MS was used to identify the metabolites. Forty metabolites including 12 amino acids, 9 organic acids, 11 sugars, 3 fatty acids, and 5 others (Table 3).

**Table 3:** Influence of the *Aspergillus aculeatus* on metabolites in leaf of perennial ryegrass exposed to 400 mM NaCl concentration.

**Metabolites**	**Treatment (0 and 400 mM NaCl concentration)**			
	**CK**	**F**	**400 mM**	**400 mM + F**
**AMINO ACIDS**				
Alanine	0.19 ± 0.032b	0.15 ± 0.013b	0.33 ± 0.054a	0.22 ± 0.088b
Valine	0.09 ± 0.017b	0.08 ± 0.008b	0.20 ± 0.032a	0.15 ± 0.051ab
Serine	0.35 ± 0.066a	0.30 ± 0.068a	0.47 ± 0.096a	0.35 ± 0.044a
Threonine	0.15 ± 0.018a	0.11 ± 0.022a	0.22 ± 0.030a	0.17 ± 0.037a
Proline	0.53 ± 0.011b	0.42 ± 0.088b	3.92 ± 0.809a	1.93 ± 0.476b
GABA	0.03 ± 0.014c	0.03 ± 0.005c	0.13 ± 0.012a	0.08 ± 0.037b
Glutamic acid	0.14 ± 0.008a	0.09 ± 0.027b	0.08 ± 0.025b	0.07 ± 0.008b
Asparagine	0.10 ± 0.011b	0.05 ± 0.021b	0.17 ± 0.069a	0.05 ± 0.044b
Tyrosine	0.03 ± 0.014a	0.03 ± 0.013a	0.02 ± 0.003a	0.02 ± 0.003a
Tryptophan	0.01 ± 0.000b	0.01 ± 0.000b	0.04 ± 0.013a	0.04 ± 0.014a
Ethanolamine	0.05 ± 0.006a	0.04 ± 0.014a	0.12 ± 0.044a	0.09 ± 0.031a
Isoleucine	0.05 ± 0.009a	0.04 ± 0.010a	UD	UD
**ORGANIC ACIDS**				
Succinic acid	0.05 ± 0.008a	0.03 ± 0.008a	0.06 ± 0.018a	0.05 ± 0.012a
Glyceric acid	0.03 ± 0.006b	0.02 ± 0.006ab	0.05 ± 0.006a	0.05 ± 0.014a
Fumaric acid	0.03 ± 0.008a	0.01 ± 0.001ab	0.02 ± 0.003ab	0.01 ± 0.005b
Malic acid	3.20 ± 0.445a	2.16 ± 0.132b	1.81 ± 0.277b	1.78 ± 0.054b
Trihydroxybutyric acid	0.02 ± 0.008b	0.02 ± 0.003b	0.05 ± 0.009a	0.05 ± 0.005a
Phthalic acid	0.02 ± 0.007a	0.01 ± 0.000a	0.01 ± 0.003a	0.01 ± 0.005a
Carboxylic acid	0.67 ± 0.255a	0.77 ± 0.185a	1.20 ± 0.357a	0.72 ± 0.105a
Citric acid	1.06 ± 0.075a	1.11 ± 0.124a	UD	UD
Glucuronic acid	0.01 ± 0.006a	0.01 ± 0.000a	0.03 ± 0.012a	0.02 ± 0.004a
**SUGARS**				
Sorbose	0.18 ± 0.000a	0.16 ± 0.090a	0.03 ± 0.003b	0.02 ± 0.004b
Psicose	0.36 ± 0.000a	UD	0.02 ± 0.005b	0.02 ±0.005b
Galactose	0.06 ± 0.011b	0.02 ± 0.010b	0.17 ± 0.030a	0.12 ± 0.053a
Glucose	0.74 ± 0.250c	0.16 ± 0.059d	2.09 ± 0.149a	1.68 ± 0.170b
Mannose	0.14 ± 0.048c	0.03 ± 0.014d	0.45 ± 0.039a	0.33 ± 0.051b
Fructose	0.77 ± 0.486b	0.24 ± 0.152b	2.33 ± 0.241a	2.00 ± 0.282a
Mannopyranose	0.01 ± 0.002b	0.01 ± 0.000b	0.02 ± 0.005b	0.03 ± 0.009a
Glucopyranose	0.01 ± 0.001b	UD	0.06 ± 0.031a	UD
Sucrose	3.74 ± 0.625b	2.85 ± 0.644b	13.93 ± 2.607a	12.44 ± 1.910a
Galactinol	0.02 ± 0.004a	0.04 ± 0.020a	0.05 ± 0.018a	0.05 ± 0.019a
Galactopyranose	UD	0.02 ± 0.007a	0.02 ± 0.002a	0.02 ± 0.010a
**FATTY ACIDS**				
Octadecanoic acid	0.10 ± 0.028a	0.08 ± 0.043a	0.09 ± 0.029a	0.08 ± 0.009a
Hexadecanoic acid	0.15 ± 0.030a	0.10 ± 0.047a	0.12 ± 0.029a	0.17 ± 0.012a
Glycerol	0.56 ± 0.108a	0.41 ± 0.147a	0.58 ± 0.138a	0.61 ± 0.092a
**OTHERS**				
Myo-Inositol	0.30 ± 0.036a	0.30 ± 0.091a	0.31 ± 0.067a	0.26 ± 0.011a
Glyceryl-glycoside	0.02 ± 0.011a	0.02 ± 0.003a	0.03 ± 0.008a	0.02 ± 0.001a
Pentasiloxane	0.03 ± 0.008a	0.04 ± 0.008a	0.02 ± 0.003a	0.03 ± 0.014a
Phosphate	0.02 ± 0.004a	0.04 ± 0.000a	0.05 ± 0.025a	0.03 ± 0.008a
Silanol	0.33 ± 0.080b	0.32 ± 0.029b	0.74 ± 0.086a	0.25 ± 0.079b

*F represents A. aculeatus. NaCl concentration is 0 and 400 mM and the results shown are the mean ± SD. The numbers marked with the same small letter indicate no significant differences for comparison of treatments based on SNK test (P < 0.05). UD, undeterminable*.

In generally, the concentrations of most of the metabolites were altered by salt treatment, and exhibited an up-regulated trend. Conversely, the application of fungi decreased a large proportion of metabolites concentrations compared with salt stress. Among the various metabolites, 6 amino acids (Alanine, Valine, Proline, GABA, Asparagine, and Tryptophan), 2 organic acid (Glyceric acid and Trihydroxybutyric acid), and 4 sugars (Glucose, Mannose, Fructose, and Sucrose) increased apparently, and 2 sugars (Sorbose and Psicose) decreased obviously under salt treatment compared to control conditions. However, in *A. aculeatus*-inoculated plant leaves, 4 amino acids (Alanine, Proline, GABA and Asparagine) and 2 sugars (Glucose and Mannose) declined notably compared to those of non-inoculated plants (Table 3).

**Figure 6:**
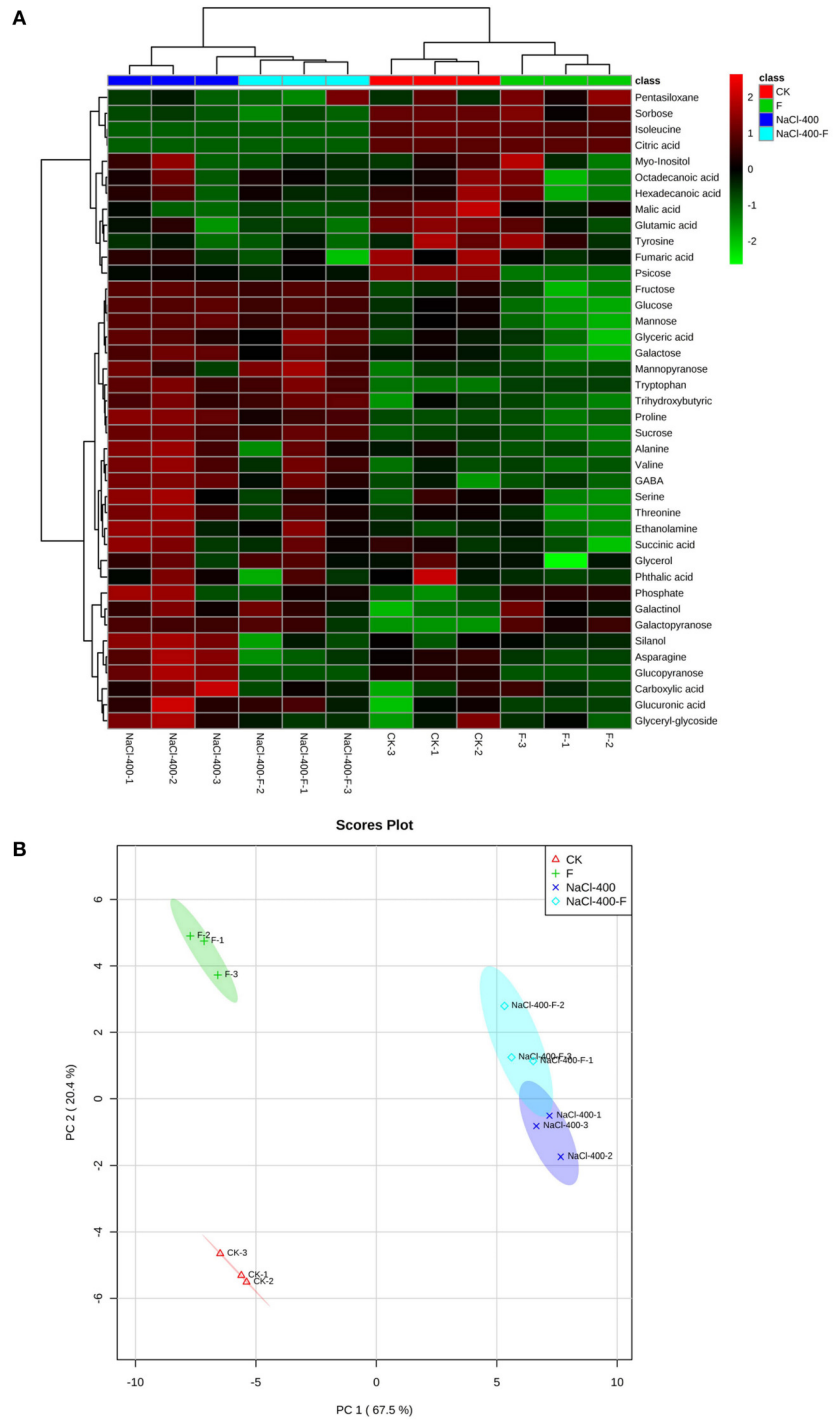
Hierarchical cluster analysis (HCA) **(A)** and principle component analysis (PCA) **(B)** of metabolites in leaves of perennial ryegrass (salt concentration is 0 and 400 mM). F represents *A. aculeatus* treatment.

Furthermore, the hierarchical cluster analysis (HCA) and principle component analysis (PCA) were applied to data sets. In HCA, it was simple to find that all samples clustered as two major groups corresponding to the salt treatments group, and without a salt group. Two subgroups were consistent with the fungi-inoculated group and their non-inoculated counterparts, which could be distinguished in the two major clusters, as shown in Figure 6A. The PCA of metabolites in plant leaves separated clearly between different treatments. In addition, the first principle component (PC1) revealed a clear separation between with and without salt treatment, which was represented 67.5% of the total variation. PC2 with 20.4% of the total variation clearly separated the fungi-inoculated samples from non-inoculated samples in the second dimension (Figure 6B).

## Discussions

This study investigated *A. aculeatus*-mediated protective mechanism of perennial ryegrass responded to salt stress. Earlier studies have demonstrated that salt-treated perennial ryegrass displayed reduced growth rate, transpiration rate, chlorophyll content, and turf quality (Hu et al., 2011, 2012). Those findings are consistent with our study which has indicated that salt exposure dramatically declined the growth rate relative to non-saline conditions. Strikingly, inoculation with *A. aculeatus* significantly alleviated the damage induced by salt treatment for inoculated plants when compared to non-inoculated perennial ryegrass. Some researchers have demonstrated that beneficial fungi, which can elevate plant performance under salt exposure (Waller et al., 2005; Baltruschat et al., 2008). In addition, plant root colonizing microorganisms that produce phytohormone such as indole-3-acetic acid to mitigate the deleterious effects of salt stress on plants (Egamberdieva, 2009). Taken together, these results, and observations suggest that *A. aculeatus* play a pivotal role in the tolerance of perennial ryegrass to salt stress, which explains the high growth rate and forage quality accompanying the better growth state in plants inoculated with the *A. aculeatus*.

To further elucidate the mechanism underlying *A. aculeatus*-mediated plant tolerance to salinity, we focused on physiological makers, such as lipid peroxidation, H_2_O_2_ accumulation, and antioxidant enzymes activities. Our data showed that salinity triggered oxidative stress in perennial ryegrass leaves as indicated by the increase in EL, MDA, and H_2_O_2_ level. The previous studies have reported that salt stress could elicit membrane damage and lipid peroxidation in turf grass (Hu et al., 2012), thereby supporting our finding. Interestingly, in our study, we found that inoculation of *A. aculeatus* dramatically mitigated the salt-induced lipid peroxidation, which was associated with a decrease in the accumulation of MDA, and H_2_O_2_. The previous study has reported that NaCl-induced lipid peroxidation was significantly attenuated in plants inoculated with *P. indica* (Baltruschat et al., 2008). These observations suggest that *A. aculeatus* can contribute to the amelioration of lipid peroxidation and maintenance of membrane functions when plants are subjected salt. Usually, tolerance of the plant to salt is associated with a high antioxidant enzymes activity (Shalata et al., 2001). Salt-induced ROS production is counteracted by a large battery of enzymatic scavengers, such as POD, CAT, SOD, or other antioxidants (Apel and Hirt, 2004). H_2_O_2_ as a kind of important ROS, produced by SOD activity, can damage cellular membrane lipids (Mittler, 2002). CAT and POD are the major H_2_O_2_ scavenging enzymes in plants and CAT is involved in scavenging H_2_O_2_ by degrading it into water and oxygen (Mittler, 2002). Our results indicated that salt stress generated the accumulation of H_2_O_2_ and increase of CAT and POD activity in perennial ryegrass. Therefore, we suggest that enhanced CAT activity coordinated with the alteration of POD activities in perennial ryegrass plays a vital protective role in the process of scavenging ROS. Distinctly, as we have observed, exogenous inoculation of *A. aculeatus* has been found to significantly decrease the CAT, and POD activities and the concentration of H_2_O_2_. Overall, these results suggest that exogenous *A. aculeatus* could contribute toward detoxification H_2_O_2_ by elevating CAT and POD activities under salt stress, and the antioxidants might be playing a crucial role in *A. aculeatus*-mediated plant tolerance to salinity.

In an attempt to shed light on the role of fungi *A. aculeatus* in plant response to salt stress, our study also focused on photosynthetic efficiency, and forage quality. Enhanced photosynthesis is accompanied with higher forage yield (Nelson et al., 1975; Peng et al., 1991). Chlorophyll a fluorescence has been proven to be an informative tool for probing the effect of abiotic stress on photosynthesis (Kalaji et al., 2011). A number of researches have illustrated the detrimental impacts of salinity exposure on plants' photosynthesis and photosystem (PSII) activity (Allakhverdiev and Murata, 2008; Kalaji et al., 2011). In agreement with those observations, in our results, an evident decline in the Chlorophyll a fluorescence transient curves was observed in salt-treated plants, compared to non-saline level. Parallel decreases in OJIP, the total carbon content of leaves also reduced when plants were subjected to salt stress (Supplementary Figure 1). Previous studies have manifested that high salinity could decrease CO_2_ acquisition by reducing stomatal conductance of leaves (Cheeseman, 1988; Abideen et al., 2014). In addition, salt stress could cause an adverse effect on the forage quality (Robinson et al., 2004), which was also observed in our study where we have observed a significant decrease induced by salinity in CP, CF, NDF, and ADF content. Previous research indicated that salinity stress caused the depression in protein synthesis (Pessarakli and Huber, 1991), which is a probably major reason for decline in crude protein content. According to those findings, we suggest that salt stress-triggered-declines in plants photosynthesis and forage quality might due to limited CO_2_ supply induced by decreased stomatal conductance and protein synthesis. To our attention, our data showed that *A. aculeatus*-inoculated plants had elevated fluorescence intensity in leaf tissue, compared to un-inoculated counterpart under similar salinity concentration. Meanwhile, photosynthetic parameters, such as φPo (F_V_/F_M_), φRo, RE_0_/RC, PI_ABS_, and PI_total_ have enhanced dramatically in *A. aculeatus*-inoculated plants under 400 mM NaCl concentration, when compared to those un-inoculated regime. Simultaneously, the enhanced total carbon content in inoculated plants was closely associated with elevated photosynthetic efficiency. Elevated photosynthesis and total carbon induced by *A. aculeatus* contributes to increase forage production, which is consistent with the previous report of Xie et al. (2014b), where the *A. aculeatus* increased plant' biomass production. Those results imply that *A. aculeatus* plays a positive regulatory role in photosynthesis of plant under salt treatment via maintaining higher growth rate, turf quality, and forage quality for inoculated plants.

One of the strategies for achieving higher tolerance to salt stress is to assist plants to re-establish ionic homeostasis in stressful conditions (Zhu, 2001; Hu et al., 2012). Previous reports have indicated that plants subjected to salt stress tend to absorb more Na^+^ ions and less K^+^ ions (Evelin et al., 2009). In addition, salinity in soil significantly decreased the absorption of mineral nutrients, especially P (Evelin et al., 2009). In correlation with this observation, our study exhibited that salt-treated perennial ryegrass remarkably enhanced the Na^+^ accumulation and decreased K^+^ and P level, compared to the non-treated plants, which is supported by many studies (Giri et al., 2007; Evelin et al., 2009; Hu et al., 2012). The decrease in mineral nutrients content such as K and P could be an explanation for declined nutritional value of perennial ryegrass accompanying reduced CF, CP, NDF, and ADF content. Previous research demonstrated that salinity could increase or decrease ionic absorption, which in turn may have a significant influence on forage nutritive value. Excessive sodium can induce deleterious influence on cell metabolism and cause an adverse effect on some enzymes. In addition, high Na^+^ accumulation also triggers oxidative stress and decline photosynthesis, thereby affecting forage quality (Mahajan and Tuteja, 2005). Different from Na^+^, K^+^ can activate a series of enzymes and plays many crucial roles in plant metabolism (Bhandal and Malik, 1988). Therefore, maintaining a proper Na^+^/K^+^ balance and high K^+^ concentration is regarded as an important mechanism of plants to cope with salt stress (Evelin et al., 2009). In our work, plants exposed to salt stress displayed higher Na^+^/K^+^ ratio than non-stressed ones. Conversely, the application of *A. aculeatus* increased K^+^ and P accumulation, significantly decreased Na^+^ concentration, thereby maintaining a lower Na^+^/K^+^ ratio and enhanced forage nutritive value in *A. aculeatus*-inoculated, compared to un-inoculated plants. Many researchers have been attempted to unravel that the positive effects of arbuscular mycorrhizal fungi on ionic homeostasis, they found mycorrhizal fungi contributed to maintaining low Na^+^/K^+^ ratio, and high K^+^ and P assimilation (Evelin et al., 2009; Ruiz-Lozano et al., 2012). According to those observations, we conclude that lower Na^+^ accumulation by *A. aculeatus*-inoculated plants further substantiates the fact that the fungi can protect the plant from the deleterious effect of excess salt ions by mitigating plant uptake of Na^+^ under saline condition. Furthermore, the increase in K^+^ as well as P uptake and a decline in Na^+^ accumulation by *A. aculeatus*-inoculated plants did not totally account for salt tolerance but at least partially it may be involved in enhancing plant resistance to salt by maintaining a low Na^+^/K^+^ ratio and ionic balance.

The metabolic homeostasis of plants was disrupted and altered by environmental changes (Widodo et al., 2009). In addition, Hu et al. (2014) found that plants exposed to salt stress would elicit a broad range of metabolic responses, and various metabolites, such as amino acids, sugars, and organic acids. It has been shown that amino acids, including proline, GABA, glutamine, and arginine among others, played a pivotal role in osmoprotection and was substantially accumulated in salt-stressed plant (Bertrand et al., 2016), which is in accordance with our findings. Among those amino acids, proline is considered as a major osmotic regulator, having multiple functions in adjusting osmosis, stabilizing the structure of proteins, and scavenging ROS (Maggio et al., 2002; Verbruggen and Hermans, 2008). For this reason, we presumed that the enhanced proline content was involved in plant protection which might in part account for the enhancement of plant tolerance to salt. What is particularly intriguing is the fact that the concentration of amino acids (such as proline, GABA, asparagine) in *A. aculeatus*-inoculated plants was obviously reduced compared to un-inoculated plant under salt exposure. Therefore, we speculate that the fungi could enhance the capacity of perennial ryegrass plant to withstand salt stress through coordinating with the amino acids exerting a protective mechanism.

On the other hand, carbohydrates (such as glucose, fructose, and sucrose) have been reported to be pivotal components for osmotic adaptation when plants respond to abiotic stress (Krasensky and Jonak, 2012). In our research, salt-induced soluble sugars were evidently accumulated in perennial ryegrass, which is consistent with the finding of Hu et al. (2013), where salt treatment resulted in an increase of soluble sugars. These results suggested that the elevated soluble sugars accumulation could contribute to the protective mechanism by adapting and adjusting the osmotic balance in perennial ryegrass against salt stress. The interesting finding of our results is that an increase of salt-induced soluble sugar, such as glucose and fructose were ameliorated in *A. aculeatus*-inoculated plant, relative to un-inoculated plants. Previous and our recent findings have confirmed proven that *A. aculeatus* could alleviate the detrimental effects induced by cadmium treatments, thereby accelerating plant growth (Xie et al., 2014a), suggesting less accumulation of sugars under stress might be due to a higher growth rate. Therefore, these results imply that the *A. aculeatus* fungi may be involved in modulating synthesis, degradation, and storage of sugars to enhance salt resistance.

In conclusion, our results showed that *A. aculeatus*, appears to confer salt tolerance of plants though altering physiological and biochemical indexes. In our study, the *A. aculeatus* was employed to accelerate plant growth and alleviate salt stress, which involved several mechanisms. According to our findings, we put forward four important mechanisms of *A. aculeatus*-mediated plant tolerance to salinity and that are respectively: (i) *A. aculeatus* can enhance plant photosynthetic efficiency; (ii) reduced the activity of antioxidant enzymes and oxidative damage; (iii) enhanced K acquisition, decreased Na accumulation, maintained the appropriate Na^+^/K^+^ ratio, and re-established ion homeostasis; (iv) regulated synthesis of metabolites and altered the concentration of metabolites (amino acids and soluble sugars). *A. aculeatus* may act as a mediator allowing plants to stimulate stress response systems, thereby enhancing plant tolerance to salt stress. Since *A. aculeatus* has no host specificity and can be propagated on a large scale in axenic culture, we highlight the high potential of this fungus in protecting plants against salt stress, which provides an effective strategy for remediating the salinity soil.

## Author contributions

XLi conceived the experiments and wrote the manuscript; XLi and SH performed the experiments and analyzed the data; GW and XLiu cultivated the experimental materials; EA revised the manuscript. YX and JF guided this experiment.

### Conflict of interest statement

The authors declare that the research was conducted in the absence of any commercial or financial relationships that could be construed as a potential conflict of interest.

## Abbreviations

ADF: acid detergent fiber
CAT: catalase
CF: crude fat
Chl: Chlorophyll
CP: crude protein
EL: electrolyte leakage
GABA: γ-aminobutyric acid
GC-MS: gas chromatography–mass spectrometry
HCA: hierarchical clustering analysis
H_2_O_2_: hydrogen peroxide
MDA: malondialdehyde
MSTFA: N-Methyl-N-(trimethylsilyl)trifluoroacetamide
NDF: neutral detergent fiber
OH^−^: hydroxyl radical
OJIP curve: F_0_, minimal reliable recorded fluorescence: at 20 ms with the pulse-amplitude modulation (PAM) fluorometer
F_J_: fluorescence intensity at the J-step (2 ms) of OJIP
F_I_: Fluorescence intensity at the I-step (30 ms) of OJIP
F_P_, maximal recorded fluorescence intensity: at the peak P of OJIP
${\text{O}}_{2}^{-}$: superoxide radical
P: phosphorus
PCA: principal component analysis
POD: peroxidase
RGR: relative growth rate
SOD: superoxide dismutase
TOC: total organic carbon.
