# Supplementary material for: The Fungus Aspergillus aculeatus Enhances Salt-Stress Tolerance, Metabolite Accumulation, and Improves Forage Quality in Perennial Ryegrass
Source: Front Microbiol. 2017 Sep 4;8:1664. doi: 10.3389/fmicb.2017.01664 (PMC5595160; doi:10.3389/fmicb.2017.01664)
Supplement: Supplementary file 1 [file Image1.PDF]

Supplementary Fig. 1

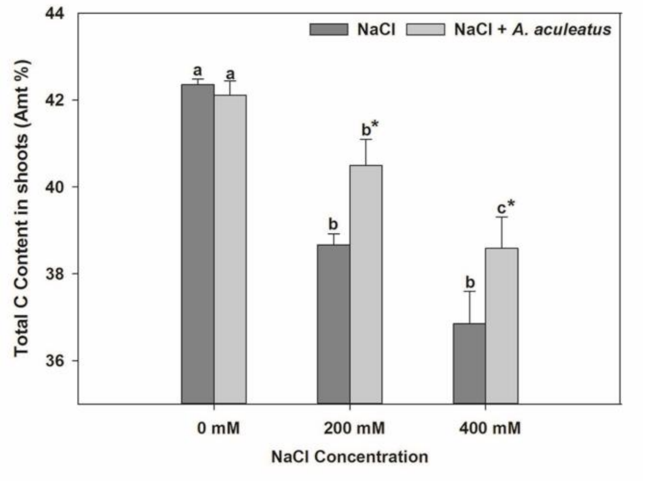

Supplementary Fig. 1 Total carbon content in leaves of perennial ryegrass exposed to salt stress. Columns marked with same small letter indicate insignificant differences under only NaCl treatment or NaCl + *A. aculeatus* treatment with the different salt concentrations (P<0.05). Columns marked with asterisk indicate significant differences under NaCl treatment and NaCl + *A. aculeatus* treatment with the same salt concentrations (P<0.05).
